# Progression of penile cutaneous horn to squamous cell carcinoma: A case report

**DOI:** 10.3892/ol.2014.2247

**Published:** 2014-06-12

**Authors:** YIHONG ZHOU, YUXIN TANG, JIN TANG, BING XIA, YINGBO DAI

**Affiliations:** Department of Urology, The Third Xiangya Hospital of Central South University, Changsha, Hunan 410013, P.R. China

**Keywords:** penile cutaneous horn, squamous cell carcinoma, quality of life

## Abstract

The current report presents the case of a 43-year-old male suffering from a penile cutaneous horn. A surgical excision of the lesion was performed and histopathology demonstrated hyperkeratosis, dyskeratosis and epithelial hyperplasia. The cutaneous horn progressed to squamous cell carcinoma <1.5 months following surgery and a partial penectomy was conducted. The International Index of Erectile Function 5 questionnaire was used to assess the patient and the score had decreased in the one-month postoperative follow-up compared with that of the preoperative period. These findings indicate that undergoing a partial penectomy on initial diagnosis of a penile cutaneous horn should be considered in order to conserve a greater quantity of the penile tissue and improve the postoperative quality of life.

## Introduction

The incidence of penile cutaneous horn has been particularly low since the first case was reported (1). It is a lesion that exhibits hyperkeratotic features on the penile glans. These hyperkeratoses are superimposed on a wide variety of benign, premalignant or malignant lesions (2). In the current report, a case of a 43-year-old male is presented. The patient underwent surgical excision of the lesion that indicated a penile cutaneous horn, this subsequently progressed to squamous cell carcinoma in <1.5 months. Finally, a partial penectomy was performed. Patient provided written informed consent.

## Case report

**Figure 1:**
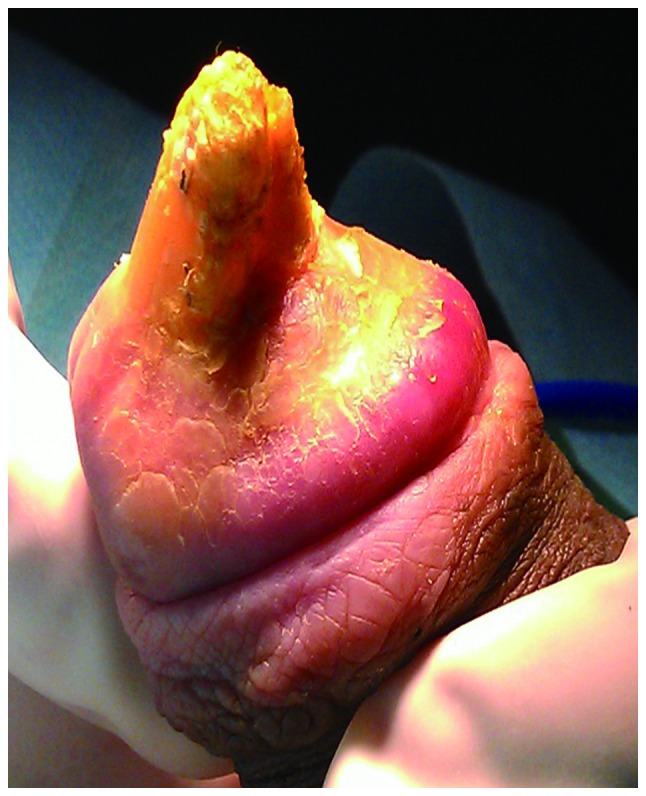
Clinical appearance of the penile cutaneous horn; a horn-like keratotic neoplasm that is yellow in colour and projects from the glans penis.

**Figure 2:**
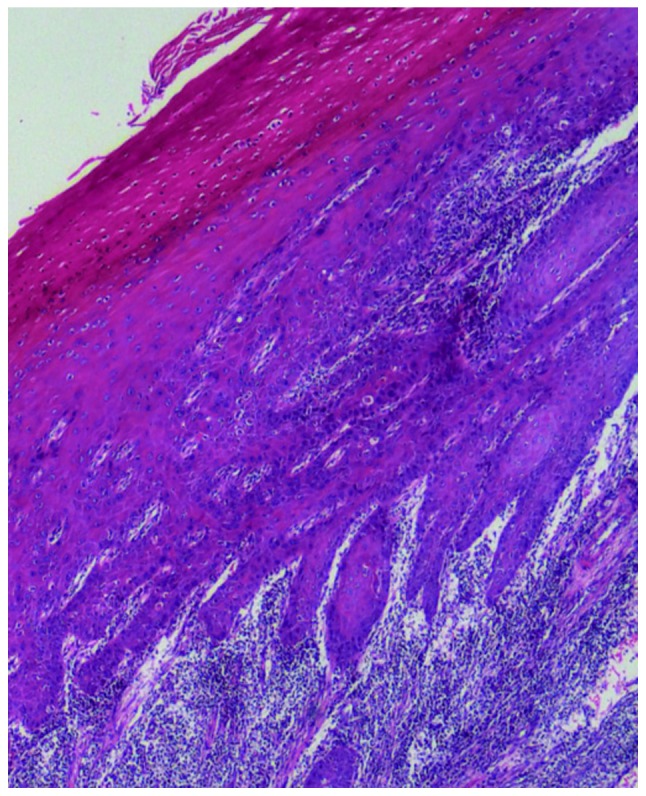
Histopathological staining of the lesion, revealed hyperkeratosis, dyskeratosis and epithelial hyperplasia (haematoxylin and eosin stain; magnification, ×40).

In February 2013, a 43-year-old male presented to the Department of Urology, The Third Xiangya Hospital of Central South University (Changsha, China) with a six-month history of a progressive growth in the glans penis. The patient had undergone a circumcision for phimosis six months previously and subsequently identified a yellow lesion projecting out from the left aspect of the glans penis. The patient experienced no pain and disregarded the lesion, which grew rapidly and prevented the patient from engaging in sexual intercourse. Examination of the patient revealed a horn-like keratinized lesion of ~2.0×1.0×0.6 cm in the glans penis with no inguinal lymphadenopathy (Fig. 1). The International Index of Erectile Function 5 (IIEF-5) questionnaire was used for assessment and the patient’s score was 18 (mild) (3). As the patient was initially reluctant to undergo a partial penectomy, a surgical excision of the lesion was performed. Histopathology the lesion demonstrated extreme hyperkeratosis, dyskeratosis and epithelial hyperplasia (Fig. 2). The patient’s postoperative recovery was uneventful.

**Figure 3:**
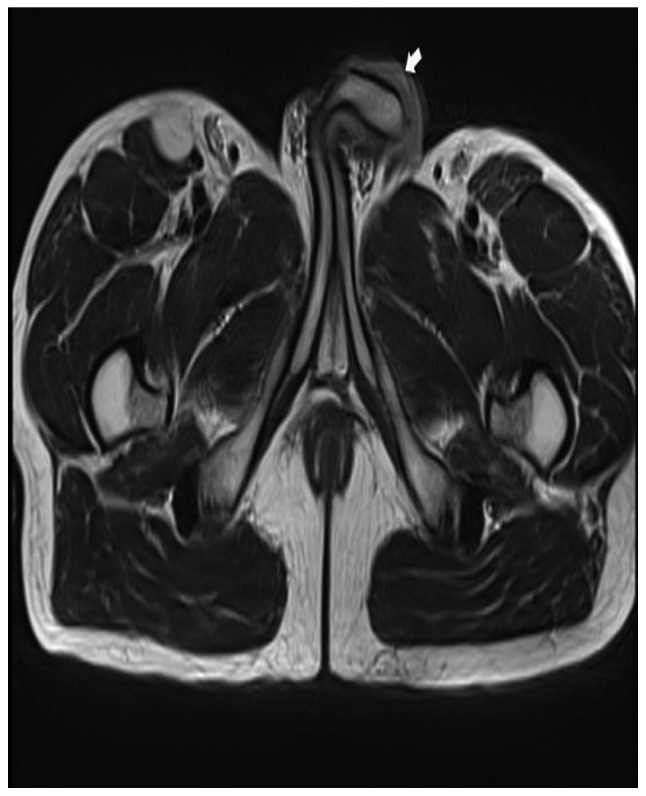
Magnetic resonance image of the pelvis. The white arrow indicates the abnormal lesion.

**Figure 4:**
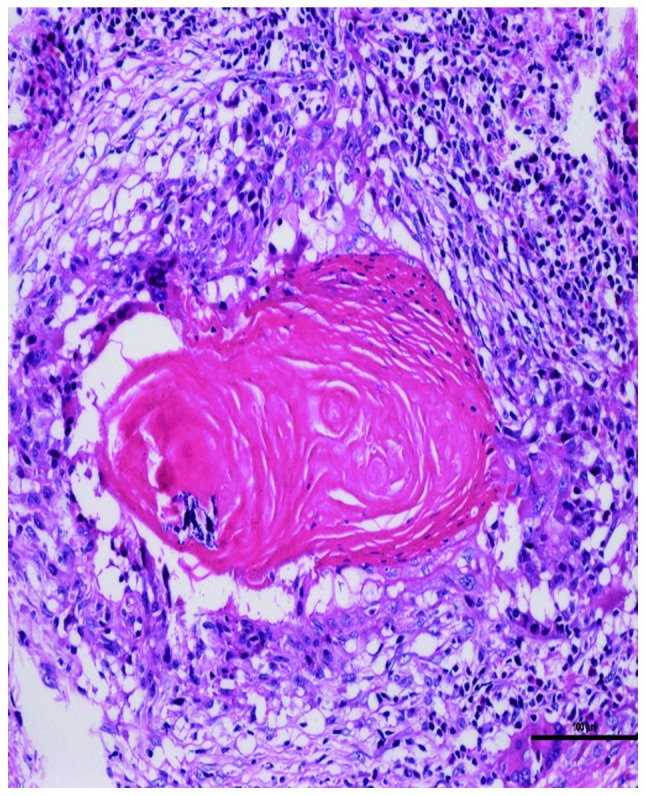
Histopathological staining identifying the lesion as a well differentiated squamous cell carcinoma (haematoxylin and eosin stain; magnification, ×200).

The first postoperative visit was conducted on day 39 following surgery, the patient presented with a painful swelling over the glans penis and purulent discharge of the urethra. On examination, the glans penis was stiff and there was a ~5-mm nodular lesion on the distal aspect of the penile shaft. Pelvic magnetic resonance imaging (MRI) revealed a lesion with an unclear boundary measuring 0.6 cm in diameter (Fig. 3). A biopsy was performed and histopathology indicated squamous atypia and suspected squamous cell carcinoma. Finally, the patient consented to a partial penectomy and histopathology revealed a well differentiated squamous cell carcinoma (Fig. 4). A six-month postoperative follow-up was conducted and the patient was able to urinate whilst standing and the IIEF-5 score was 15 (moderate). Follow-up of the patient is ongoing.

## Discussion

The etiology of penile cutaneous horn remains uncertain since the first case was reported in 1854 (1). Viral and non-viral factors may be implicated in penile cutaneous horn formation. Solivan *et al* (4) identified a positive human papillomavirus (HPV) reaction for HPV 16 using *in situ* DNA hybridization; furthermore, Zhu *et al* (5) demonstrated that the HPV 16/18 infection was positive in a patient exhibiting a penile cutaneous horn. These findings indicate that the HPV infection may be one of the pathogens involved. In addition, the presentation of a penile cutaneous horn may be associated with phimosis, warts and trauma, amongst other conditions (6,7). To the best of our knowledge, penile cutaneous horn usually occurs following an adult circumcision (2). However in the present case, the formation of the penile cutaneous horn preceded the patient’s circumcision, as it was during the circumcision that the lesion was identified. It was hypothesized in the present study that continuous, chronic preputial inflammation aggravates the formation of a penile cutaneous horn and the trauma of circumcision accelerates its development.

The European Association of Urology guidelines on penile cancer classify penile cutaneous horn as a premalignant lesion (8) and approximately one-third of penile cutaneous horns are associated with an underlying malignancy (2). It has previously been reported that MRI is helpful when there is uncertainty regarding the depth of infiltration or proximal extension (9). When the patient in the present case was hospitalized for the second time, a physical examination presented a nodular lesion on the distal aspect of the penile shaft and the MRI result provided the basis of the nature of the lesion.

As instances of penile cutaneous horn are particularly rare and the majority of studies regarding them are case reports, various treatment methods have been adopted, including laser therapy, administration of keratolytic agents and surgical excision (10,11). In the present report, a malignant lesion did not exist when the patient was first diagnosed with a penile cutaneous horn. However, a malignancy was present less than 1.5 months following surgery, thus a partial penectomy was conducted. The IIEF-5 questionnaire, which has previously been used to evaluate sexual function and satisfaction following a partial penectomy (12), was used in the present case; the patient’s IIEF-5 score in the preoperative period was greater than the score six months postoperatively. Based on these findings, undergoing a partial penectomy when a penile cutaneous horn is initially diagnosed should be considered in order to conserve a greater quantity of penile tissue and improve the postoperative quality of life.

In conclusion, to the best of our knowledge, such a rapid progression of a penile cutaneous horn to squamous cell carcinoma has not previously been described. In addition, a decline in the patient’s quality of life was noted. Thus, performance of a partial penectomy should be considered as soon as a diagnosis of penile cutaneous horn is determined.
